# Pig production in Africa: current status, challenges, prospects and opportunities

**DOI:** 10.5713/ab.23.0342

**Published:** 2024-02-23

**Authors:** Akinyele O. K. Adesehinwa, Bamidele A. Boladuro, Adetola S. Dunmade, Ayodeji B. Idowu, John C. Moreki, Ann M. Wachira

**Affiliations:** 1 Livestock Improvement Programme, Institute of Agricultural Research & Training, Obafemi Awolowo University, PMB 5029, Moor Plantation, Ibadan, Nigeria; 2 Department of Animal Science, Faculty of Animal and Veterinary Sciences, Botswana University of Agriculture and Natural Resources, P/Bag 0027, Gaborone, Botswana; 3 Kenya Agricultural and Livestock Organisation Non-Ruminant Institute, POB 169-50100, Kakamega Kenya

**Keywords:** Farmers, Policy, Pork, Smallholder, Transformation, Value-chain

## Abstract

Pig production is one of the viable enterprises of the livestock sub-sector of agriculture. It contributes significantly to the economy and animal protein supply to enhance food security in Africa and globally. This article explored the present status of pig production in Africa, the challenges, prospects and potentials. The pig population of Africa represents 4.6% of the global pig population. They are widely distributed across Africa except in Northern Africa where pig production is not popular due to religio-cultural reasons. They are mostly reared in rural parts of Africa by smallholder farmers, informing why majority of the pig population in most parts of Africa are indigenous breeds and their crosses. Pig plays important roles in the sustenance of livelihood in the rural communities and have cultural and social significance. The pig production system in Africa is predominantly traditional, but rapidly growing and transforming into the modern system. The annual pork production in Africa has grown from less than a million tonnes in year 2000 to over 2 million tonnes in 2021. Incidence of disease outbreak, especially African swine fever is one of the main constraints affecting pig production in Africa. Others are lack of skills and technical know-how, high ambient temperature, limited access to high-quality breeds, high cost of feed ingredients and veterinary inputs, unfriendly government policies, religious and cultural bias, inadequate processing facilities as well as under-developed value-chain. The projected human population of 2.5 billion in Africa by 2050, increasing urbanization and decreasing farming population are pointers to the need for increased food production. The production systems of pigs in Africa requires developmental research, improvements in housing, feed production and manufacturing, animal health, processing, capacity building and pig friendly policies for improved productivity and facilitation of export.

## INTRODUCTION

Africa’s vast landmass and its increasing population has contributed to global livestock production. Africa is the second largest continent in the world, it has a population of about 1.4 billion, representing 17.9 percent of the world’s total population [1]. The extraordinary geographical and biological diversities, as well as, the socio-cultural richness of Africa play a central role in shaping the global livestock industry. Africa’s livestock population constitutes one-third of the global livestock production [2] and accounts for around 40% of Africa agricultural GDP [3]. Livestock will become increasingly significant in Sub-Saharan Africa (SSA) in the future, as the demand for animal-sourced food rises due to rising population, income, and urbanization. Low- and middle-income consumers are projected to need 107 million tonnes more meat and 5.5 million tonnes more milk by 2050, than they did between 2005 and 2007 [4]. The per capita annual consumption of meat and milk in SSA is estimated to be 14 kg and 30 L, respectively, by 2050 [2].

Pig production is one of the avenues through which Africa can meet its need for animal protein and also contribute to global needs through export. Africa contributes 1.67% of the global pigmeat production. Pig production is an important aspect of the livestock sub-sector of Agriculture in Africa. The contribution of pig production to the sustenance of livelihood in the continent through the provision of nutritious source of protein, job and means of generating income cannot be overemphasized. Pork has high biological value, contains essential amino acids and it is easily digestible. Pork shared the nutritional qualities of red and white meats, thus; it is regarded as “pink meat” [5]. The global trend in world meat production (2016–2020) and consumption has shown that pig meat is a very important source of animal protein in human diets. Estimates from FAO data [5,6] shows pork as the world’s most widely eaten meat, accounting for 36% of the world total meat consumption, surpassing poultry (33%), beef (24%) and, goats and sheep (5%). However, pig meat is not the most consumed meat in Africa, it ranks after poultry, beef, mutton and chevon respectively.

Pig rearing is done mainly under extensive system of production in most parts of Africa, as they are left to scavenge for food, with provision of little care for the animals [7]. It is however rapidly moving from the traditional extensive system to semi-intensive and intensive systems of production. Inherent traits of pigs such as high fecundity, short generational interval, better feed conversion indices and early maturity when compared to other livestock species has identified pig as an animal with potential to contribute immensely to enhancing food and nutrition security in Africa and globally. Investment in pig production has proven to be one of the most profitable livestock businesses because of its relatively low cost of production compared to other major livestock farming businesses [8]. Hence, pig farming is a buoyant sector with good potential to bridge the gap that exist in animal protein supply while providing affordable sources of meat as well as contributing to Africa and world economy, through job creation and income generation. Despite the potentials in pig production in Africa, there are several constraints facing the sector, that is preventing it from being fully harnessed. Hence, this paper aimed to examine the current status of pig production in Africa with respect to its production system, population distribution, pork output and per capita pork consumption, challenges and prospects.

## CURRENT STATUS OF PIG PRODUCTION IN AFRICA

### Pig production systems in Africa

Pigs are widely distributed all over Africa with more than 70% of its population found in rural communities [9], where in most cases, the pigs are allowed to scavenge for food. Also, West Africa has the highest pork production, with Nigeria contributing 65%, and having 90% of its pig population raised under extensive system [10]. Pig production is a means of livelihood in many parts of Africa, especially for women and youths, who are involved in small scale farming. In Africa, pig production systems are diverse and complex among countries, each with its own set of challenges and opportunities. Globally, the pig industry was affected by incidence of disease outbreaks, especially Africa swine fever (ASF), as well as, increases in cost of inputs, which has affected production cost and output. These have affected Africa due to the prevalent production systems.

The production systems of pig in Africa often range from traditional scavenging, smallholder systems, to more intensive commercial setups [11,12]. The traditional system allows the pigs to roam around to scavenge for food and as such are exposed and become vulnerable to the threats of extremes of weather and disease infection, as well as, theft. Animals reared under this system are mainly indigenous breeds. Crosses between the indigenous and exotic breeds are also used in this system, but they do not reach market weight easily. This is because traditional system may not provide adequate nutrition for optimal growth, while the scavenging activities utilizes energy that is supposed to be used for growth and development [8]. Scavenging animals are usually associated with high rate of disease infection.

The intensive system, is based mainly on the exotic breeds, and their crosses. However, they require use of formulated diets, which can be expensive, due to the high cost of imported feed ingredients and scarcity of grains, which is required to meet the nutritional needs of the animals for optimal productivity in confinement. Intensive system of production also involves provision of good housing, health services and waste disposal facilities for proper management, thus, requiring more capital. While some pig farmers in Africa are investing in the use of modern pig farm equipment, many, especially the smallholder farmers use locally sourced materials such as canes, bamboo, planks or mud for wall or demarcation of the house. The indigenous breeds of pigs and their crosses are the common breeds reared in the traditional system, though not exclusive. The exotic breeds, such as Large white, Landrace, Duroc, Hampshire and Pietrain are common in the intensive system of production [8], even though Large White, Landrace and their crosses are more prevalent. The traditional systems with its open scavenging and minimal biosecurity measures facilitates disease spread [11]. Intensive systems, if managed well, can implement biosecurity measures to mitigate disease risks. However, the cost of implementing biosecurity measures may be a challenge for small scale pig farmers in Africa [11].

Recently, there have been establishment of clusters and pig farm estates for smallholder farmers in Africa. In some instances, they are organized into farmer groups or cooperatives. This is to grant smallholder farmers easy access to some facilities large scale farmers also enjoy. As a member of a cluster or cooperative, the farmers have easy access to facilities such as credit, government interventions, procurement of inputs on wholesale to share among members, veterinary services, good infrastructure and easy access to market. A good example is the Oke Aro Pig Farm Cluster in Lagos State, Nigeria, which is the largest pig farm estate in West Africa [13]. A major challenge of this system is the inability to consistently implement and enforce good biosecurity measures. Farmers and farm attendants compromise biosecurity protocols through sharing of equipment and labour among different units in the cluster, thereby facilitating easy spread of diseases and re-occurrence.

Over the years, pig production in Africa have been influenced by a combination of factors, including technological advancements, increased awareness of sustainable practices, changing consumer demands and perception, as well as efforts to address challenges like disease management and food security [12]. There has been progress in developing pig breeds that are well-adapted to local conditions and exhibit improved disease resistance, growth rates, and reproductive performance. South Africa and Nigeria appears to have the highest pork yield per animal across Africa [1] and this may be as a result of the use of improved breeds and adoption of intensive or modern production system.

### Pig population distribution in Africa

Pig population in Africa as at 2020 was 44 million, this is approximately 4.6% of the total world pig population [1]. Among the five regions in Africa, Eastern Africa has the highest proportion of pig population, representing 38% of the total pig population in Africa (Figure 2). Northern Africa has the least pig population, contributing less than 1% of the total Africa pig population, and this can be attributed to the cultural-religious demography of the region, which does not support the consumption of pork, thus affecting the per capita consumption and production of pork in the region. Other regions including Western, Central and Southern Africa are prominent in pig production, contributing approximately 33%, 17%, and 12%, respectively, of continental pig population [1].

**Figure 1:**
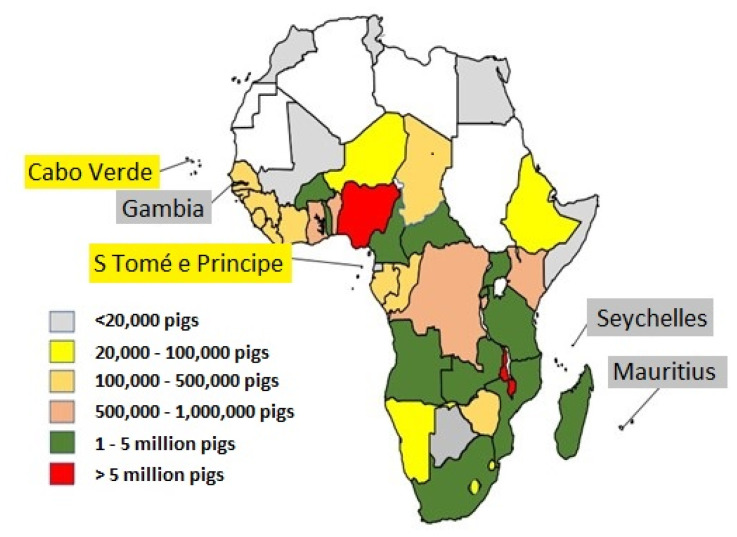
Pig population distribution across different countries of Africa [35].

**Figure 2:**
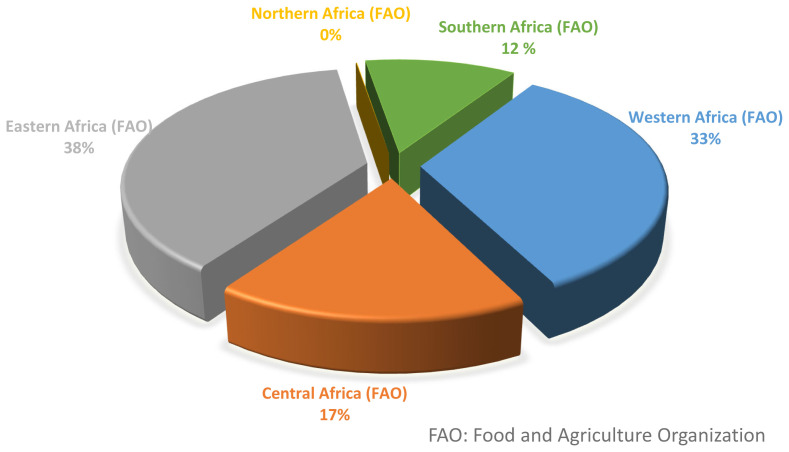
Distribution of pig population in different regions of Africa [9].

Nigeria and Malawi are the two countries in Africa whose pig population is above 5 million (Figure 1), with actual figure exceeding 7 million heads (Table 1). Together, both countries contribute approximately 35% of the total pig population in Africa. While Nigeria has the highest pig population, Malawi has the highest pig population density in Africa.

**Figure 3:**
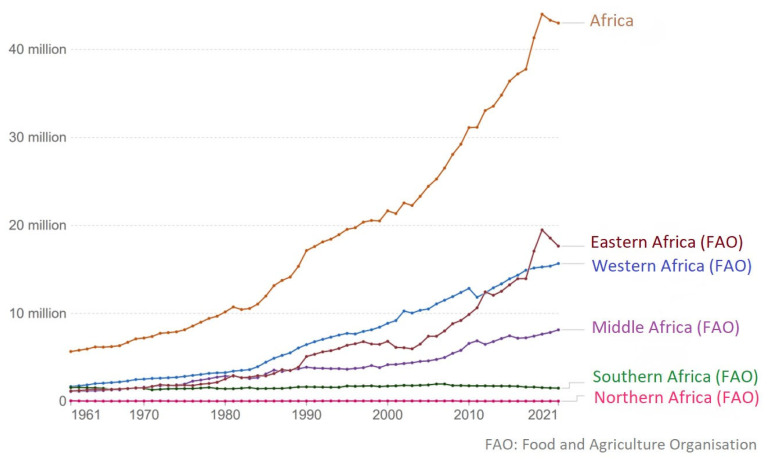
Trend of pig population in Africa: 1961 – 2021 [9].

As indicated in Figure 3, pig population in Africa has been increasing over the years, especially, from the year 1980. This growth can be attributed to increased standard of living and population growth, which is creating need for more food. While there has been increased growth in the pig population of Eastern, Western, and Middle (Central) Africa, the pig population in Northern and Southern Africa are exceptions. The pig population in these two regions have not increased much over the years. Between 2001 and 2021, Africa pig population has increased from 21 million to 43 million, which is about 101% growth in the pig population of the continent.

### Pig meat yield in Africa

**Figure 4:**
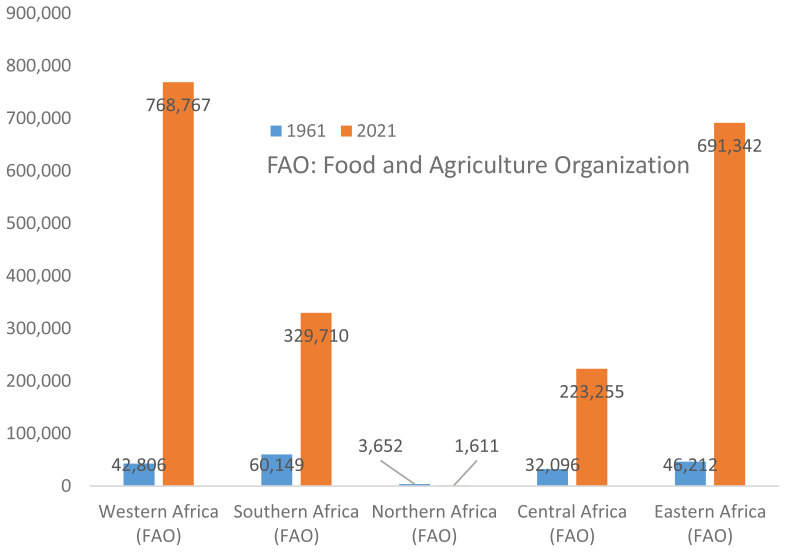
Total pork production (tonnes) in different regions of Africa, 1961 and 2021 [9].

**Figure 5:**
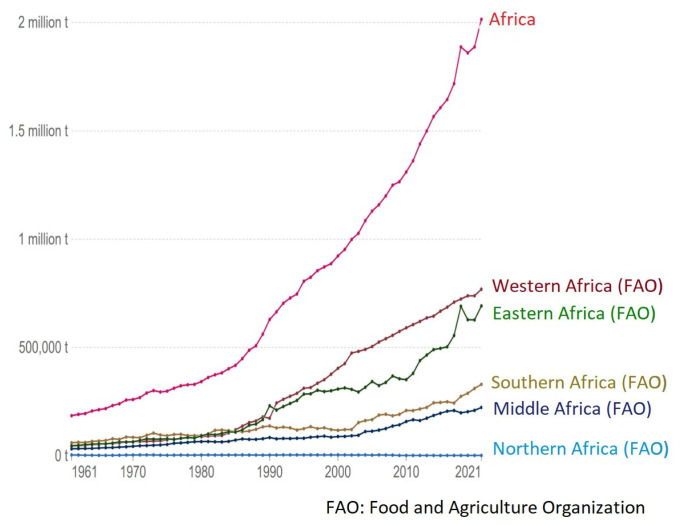
Africa pig meat production (tonnes), by region [1].

The total pork production in Africa in 2021 was over 2 million tonnes [1]. The pork production (tonnes) in different regions of Africa between 1961 and 2021 is as presented in Figure 4. Pork production is shown to have increased in the different regions of Africa over the years (Figure 5), except in Northern Africa, with 56% decrease in pork production. The rate of pork production increase across other regions were 1,696%, 448%, 596%, and 1,396% in Western, Southern, Central, and Eastern Africa, respectively.

The total pork production of Western Africa in 2021 was 768,766.80 tonnes. South Africa had the highest per capita consumption (4.19 kg) of pork in Africa and it is a major producer of pork in Southern Africa. As at 2021, its contribution to pork production in Southern Africa region was approximately 320,450 tonnes, representing 97% of the 329,710 tonnes of pork produced from the region (Table 1). The remaining 3% was contributed by other countries within the region, which majorly include Namibia, Eswatini (Swaziland), Lesotho and Botswana.

The pattern of pork production in Northern Africa over the years has been undulating, with no substantial growth. The sharp drop in the pork output in this region from 2008 [1] can be attributed to decreased pig population, which arose from what can be termed as malicious culling of pigs, which claimed that pigs are reservoirs of avian influenza viruses following the outbreak of the disease in poultry in the region. Pork production dropped from 3,893 tonnes in 2006 to 1,328 tonnes in 2011, and further down to 1,234 tonnes in 2015 [1]. However, the production figure in the region rose again to 1,610 tonnes in 2021, and it is expected to continue to improve as some local farmers are showing interest in producing pork for tourist and foreigners that are resident in the region. In Egypt, pig production is concentrated among the Coptic Christian minority who are just about 10% of the population. However, contribution of countries such as Libya, Sudan, and Western Sahara to pork output in this region is highly negligible.

In Morocco, most of the pig farmers are poultry producers who ventured into pig production after their poultry farms were affected by waves of bird flu. Their target is usually to produce pork for visiting tourists and some foreigners that are resident in the country, as well as, to also bridge the gap on importation of pork, mostly from Spain, Belgium and France, into the country. Morocco has also been reported to export pigs to countries, such as Gambia and Cyprus, resulting from the notable growth in the pig industry experienced from around 2008 to 2013 with the entrance of new local farmers, producing for consumption of resident foreigners and tourists. However, pig production in Morocco has since then not experienced much growth, with the approximate pig population of 8,000 pigs and production of 630 tonnes of pig meat per annum at 2021 [6].

### Per capita pork consumption in Africa

The consumption of pork varies widely across different African countries due to cultural, religious, economic, and geographical factors. Pork is not the most consumed meat in Africa. The per capita consumption of pork in Africa (1.53 kg) ranks after poultry (6.30 kg), beef (5.22 kg) and, chevon and mutton (2.39 kg). The per capita pork consumption in Africa (1.55 kg) is not comparable to 14.35 kg in Asia and 33.79 kg in Europe. The level of meat consumption increases with increased prosperity or income, as taste and preferences change. Like in some other parts of the world, the consumption of pork in Africa is largely affected by religious and cultural beliefs, that prohibits the consumption of pork.

Southern Africa has the highest per capita pork consumption (3.99 kg) in Africa, while the Middle, Western, Eastern, and Northern Africa have per capita pork consumption of 2.18, 1.97, 1.38, and 0.01 kg, respectively [1]. South Africa has a diverse population, with a range of dietary preferences, and pork is commonly consumed there. However, in countries with larger Muslim or Jewish populations, pork consumption tends to be much lower. These religious diversities have significant impacts on pork consumption patterns in various African nations. Countries with a majority of Muslim population, such as Egypt, Tunisia and Algeria, typically have low per capita pork production and consumption due to Islamic dietary restrictions [14]. As at 2020, Egypt produced about 628 tonnes of pork, which is lower, when compared to over 302,000 tonnes and 311,000 tonnes produced in Nigeria and South Africa, respectively [1]. Urban areas experiencing economic growth are known to shift towards more diverse diets, with observed alternative protein sources. This shift may have considerable impact on pork consumption. The spread of international fast-food chain, found in most African urban centers, including those that offer pork-based products, has been documented [15].

### Marketing

Although pig marketing channels in Africa are found to be simple, as live animal sales constitute the primary marketing channel for the sales of pigs by producers [13]. The conditions for transportation, handling, and selling of pigs are subject to many challenges [16]. Most farmers are forced to market live animals to middlemen who, in turn sell to butchers. At times the farmers slaughter by themselves and sell to the final consumer as fresh pork, to maximize profit. This usually occur when the middle men are trying to force the price down, to take advantage of farmers who are under pressure to sell to augment cost. However, there are out-growers system as well, where farmers produce for targeted buyers or processors, who uptake the animals for processing into products. There are a number of constraints to the efficient functioning of the market, arising from lack of market information, limited capital, lack of access to formal credit sources, lack of good roads, and exorbitant transport fees. These constraints increase actual market and transaction costs. Non availability of state support for pig products marketing, as we have in most part of the world is also experienced in most part of Africa. Therefore, government support in terms of infrastructure and policy would enhance pig marketing.

## CHALLENGES OF PIG PRODUCTION IN AFRICA

### Common challenges

The challenges of pig production in Africa are diverse and it varies from country to country, and even within regions. Some common challenges encountered in pig farming across Africa are as listed below:

#### i. Inconsistent policies and programmes within countries and across the continent

South Africa is a major player in Africa’s pork industry and engages in pork exports. If export requirements, particularly sanitary and phytosanitary standards, are inconsistently communicated or abruptly altered, it can lead to delays in shipments, rejection of products, and damage to trade relationships. In the last decade, countries like Kenya have experienced fluctuations in import regulations for pork products. The implication of inconsistent import policies, changing frequently between strict bans and relaxed rules, impact the stability of the local market. Importers and traders struggle to navigate these changes, leading to supply disruptions and price volatility.

**Figure 6:**
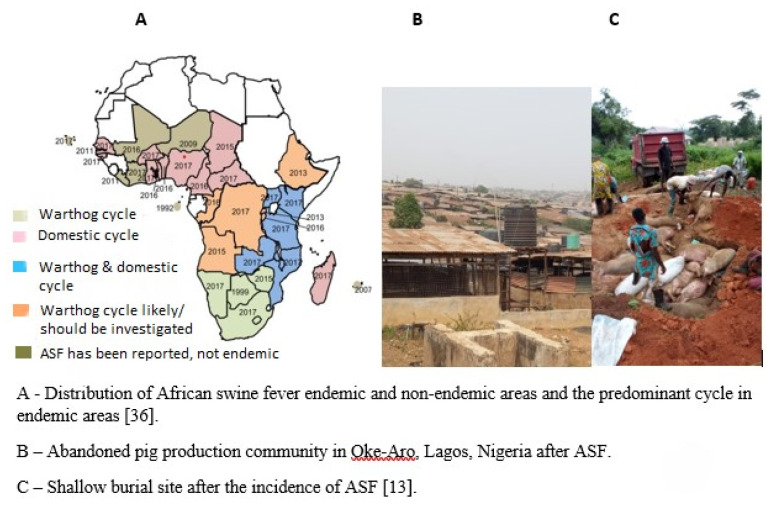
Epidemiology and effect of African swine fever in Africa [13].

Inconsistency in disease control policies in some African countries is also a major challenge facing pig production. For example, ASF outbreaks have been a significant challenge for pig production in Africa and globally (Figure 6). About 25% of global pig population was lost to ASF attack between 2018 and 2019. It is a highly contagious viral disease that affects domestic and wild pigs. The disease can cause high mortality rates, leading to significant economic losses and hampering pig production in affected areas, thereby resulting in a decline in pig populations and impacting the livelihoods of farmers [13]. Many farmers have left the business, arising from the re-occurrence of this disease, as there is little or no compensation for affected farmers. This has caused most farmers to keep to themselves when affected by ASF and silently market the infected animals, thereby spreading the disease further. Inconsistent policies regarding disease control measures, many at times, has led to confusion among pig farmers and makes disease prevention and control difficult.

#### ii. Limited access to high quality breeding stock

Access to high-quality breeding stock, such as genetically improved breeds and superior genetics, are limited in most African countries. This has influenced the productivity, and it is responsible for the wide variation in pig meat yield between countries like South Africa, that uses mostly improved breeds or hybrids for production and others, who rear mainly non-distinct breeds. Access to good breeds can impact the productivity and efficiency of pig farming systems, and the overall profitability of the pig industry.

#### iii. Inadequate infrastructure

Insufficient infrastructure, including proper housing facilities, cold storage facilities and processing plants, waste management systems, and transportation networks, can hinder the development of the pig industry. These infrastructural limitations make it difficult to efficiently transport pigs and pork products, resulting in increased costs and limited market access for farmers.

#### iv. High and unstable cost of inputs, such as feed and drugs

The availability and affordability of quality pig feed and medications are major challenges in Africa. The cost of commercial feed, especially when it contains imported ingredients, can be high. Inadequate knowledge of the nutrient requirements of pigs, as well as, poor knowledge of feed formulation is typical among pig farmers in Africa. This knowledge gap influences the profitability of pig farming, as pig typically require a balanced mix of energy, protein and other essential nutrient sources. Indigenous pigs which can subsist on low quality feed need not be fed high quality diet. However, imported breeds and hybrids require high quality feed. Good branded or commercial feeds, specifically for pigs, are rarely available in most countries in Africa. Availability of good feed or feed ingredients, coupled with good breed, will drive increased pork yield in Africa. Additionally, inadequate storage facilities and infrastructure for drugs and medication, as well as, feed ingredients can lead to feed spoilage, thereby affecting the effectiveness of the materials.

#### v. Limited veterinary services

Access to quality veterinary services, including vaccination programs, disease diagnosis, and treatment, is often limited in many parts of Africa [17]. This can hinder disease control and prevention efforts, leaving pig farmers vulnerable to disease outbreaks and leading to significant economic losses. Implementing effective biosecurity measures to prevent the spread of diseases is crucial in pig farming. However, limited awareness, inadequate resources, and poor biosecurity practices, can make it challenging to control disease outbreaks and maintain healthy pig populations.

#### vi. Lack of Technical knowledge and skills

Limited access to training and technical knowledge about modern pig farming techniques and its adoption have continued to be a challenge for small-scale pig farmers, who form the majority in most countries. The lack of expertise, in areas such as breeding, nutrition, artificial insemination, housing, and biosecurity practices has hampered productivity and limited the ability to adopt improved farming techniques, hence, decreased productivity and efficiency.

#### vii. Cultural and market factors

Cultural preferences and religious beliefs have affected pork consumption and market demand in some African countries. Preferences for other types of meat and cultural taboos surrounding pork consumption has limited market opportunities for pig farmers. As well, limited market access and underdeveloped value chains have posed challenges for enhanced growth of the pig industry. This is because difficulty in reaching markets, inadequate processing and storage facilities, as well as, weak market linkages have affected the profitability and sustainability of pig production.

### Climate challenges

The increase in greenhouse gas (GHG) production and concentration in the atmosphere are connected to high ambient temperature, increasing variability in precipitation pattern and other extremes of weather conditions [18,19]. These changes and associated variations in climatic conditions over time, termed as climate change affects the productivity of livestock species, including pigs. Invariably, livestock are also implicated in generating 14.5% of the total anthropogenic GHG emissions, while pig production and related activities contribute 10.1% of the total livestock contribution to GHG production [18,19]. Africa agriculture, including pig production, is sensitive to climate variability because of over-reliance on rain-fed agriculture [20], thus making it one of the regions in the world that is most vulnerable to the detrimental effects of climate change [21,22].

Water availability and usage for livestock and crop (feed) production are predicted to be affected by climate negatively [23,24]. Water needs per pig for consumption and cooling, especially, as a means of ameliorating the effect of increased temperature, is increasing, while water availability is reducing. The reduction in water availability likewise, is negatively impacting plant-based feed resources supply, especially grains, thereby causing scarcity and increase in unit price of the commodities. The challenge of climate change on pig production is aggravated by incidence of drought and desert encroachment in some parts of Africa. Namibia, for example, has been experiencing drought, which has negatively affected pig and other agricultural production activities in the country [21,25].

Climate change has been identified as a major cause of increasing ambient temperature, which consequently leads to thermal stress and tends to have a more severe implication on the performance of pigs, than cold stress, especially in Africa. Heat is an important stressor of pig [26]. Heat stress is caused by high ambient temperature, coupled with high humidity, and it compromises productivity in pig production by affecting growth rate, reproduction efficiency, as well as, its negative implications on the health status of animals and mortality.

Breeding for high adaptability and heat tolerance, using indigenous breeds of pigs in Africa is an important strategy that can be explored, to ameliorate the effect of climate change on pig production in Africa. However, animals may not be able to fully adapt to climate stressors themselves, thus, adequate cooling systems and management strategies are required to mitigate the effects of heat stress on pig production in Africa [18,26]. Forecasting seasonal climate variations for farmers and transmitting the information to them in good time can enable farmers to make climate-smart farm decisions, to avoid losses, and also, enhance grain production and supply. Pigs are affected by climate change and they also enhance it through GHG production. As such, proper management strategies to mitigate the effect of climate change on pig and minimize generation of GHG from pig production and related activities, are both important in enhancing pig production in Africa [18].

## PROSPECTS, OPPORTUNITIES AND POINTER TO BETTER FUTURE OF PIG PRODUCTION IN AFRICA

### Prospects and opportunities of pig production in Africa

Pig production in Sub-Saharan Africa presents various opportunities and potentials that can contribute to economic development, food security, and improved livelihoods for local communities. To realize the full potential of pig production in Sub-Saharan Africa, it is crucial to focus on developing a robust and sustainable pig value chain.

#### i. Food security

Pigs are highly prolific and convert feed to edible meat, better than most livestock species. This makes them a suitable animal for production of meat in quality and quantity, especially to solve the challenge of malnutrition, arising from low protein supply, which is prevalent in most parts of Africa. Also, pig meat is a rich source of protein, hence, raising pigs for meat can contribute to diversifying protein sources in diets, thereby giving consumers meat options and enhancing food security.

#### ii. Employment opportunities

The pig production value-chain which include breeding, management, processing, and marketing, can create employment opportunities for skilled and unskilled labour of the rapidly growing population of Africa. These range from farmers and technicians, to traders, farm manager, specialists in different aspects of animal production, processors, input suppliers, artisans, facility managers, logistic operators, retailers in local markets, among others. Pig production activities can be practiced by women and youths, due to the relatively low space and initial investment requirements, compared to other livestock species [27].

#### iii. Income generation and foreign exchange earnings

Pig production enterprise is a highly productive enterprise with good returns on investment. Pig production has the potential to significantly impact per capita income in Sub-Saharan Africa by providing a steady source of income for small-scale farmers. The fast reproduction rate of pigs, high growth rate and short generation interval, allows for regular sales of piglets and pork, contributing to a consistent stream of income [28]. While pigs are produced in most countries in Africa to nourish their populace, it can also serve as means of foreign exchange earnings by exporting it to neighbouring countries or outside the continent. The recent gap in the supply and demand for pork in global market is an indication of potential of foreign exchange earnings from pig production. The projected increasing demand for pork products due to population growth and changing dietary habits points to potential for increased income generation through pig production [28].

#### iv. Investment opportunities

The inherent reproductive and growth potentials of pigs, as well as, the improvement in techniques used in pig production over the years has made investment in pig production rewarding. Although the pig industry in Africa is developing, the low level of investment in technologies and infrastructure have slacked it off, from maximizing its productivity. Also, urbanization and changing dietary preferences have led to an increased demand for meat in many developing countries [13]. This presents opportunities for farmers to meet local market needs, as well as, potentially explore export markets for pork products. Investment opportunities in pig production in Africa cut across every aspect of its value chain, which is not limited to breeding, housing, feed production, animal aggregation, processing or value addition, cold storage, transportation, proper retailing outlets among others. The opportunities in pig value addition in Africa is enormous, but has not been properly tapped. Processing pork into cured products, sausages, and other value-added items can lead to higher profit margins, enhancing the overall income of farmers and processors. This presents farmers the opportunity to connect to both local and regional markets, thereby boosting trade and economic activity. As demand for pork products increases due to population growth and changing dietary habits, there is potential for further income growth [29].

The prospects of pig production in North African countries lie in the increasing demand for pork, driven by the attraction of tourists into the country and identifying pig production as an opportunity for foreign exchange earnings, by exporting it to nations with increasing demand for pork.

### Pointers to better future

The human population of Africa is projected to reach nearly 2.5 billion in 2050 [30] and this population needs to be fed. This is an indication of more demand for food, which includes pig meat. The rural-urban migration has contributed to reduced farming population and thus the need for modern or mechanized method of pig farming for increased productivity and output, in answer to rising demand for pork. Recently, there have been increased investment by private and government institutions. With good agricultural land, quality input and good infrastructure, Africa can turn into a hub for supplying pork to the world. Afterall, in 1943, Nigeria had the largest pig farm in the world, established by the United African Company (UAC), and located in Kano. The farm grew into a very large-scale farm in the ‘50s and ‘60s, during which time the pig meat was transported by rail to Lagos, at that time, the commercial city and political capital of Nigeria, before its final export out of the continent. It is unfortunate that the pig farm eventually folded up in the late ‘70s due to religious prejudice [13].

Increased urbanization, population growth and rising incomes are factors driving increased demand for meat, including pork [31]. These increased demands are pointers to need for more investment in pig production. For example, the rapid population growth and urbanization being experienced in East African cities and in a country like Nigeria, offer a large and growing market for pork products. This presents opportunities to cater for the increasing urban demand for meat products, including pork. Coordinated efforts can ensure or guarantee both safe and legal trade for pigs and pork products, for the benefits of both producers and consumers.

Enhancing pig production in Africa to meet the needs of the increasing population also requires transforming the industry from the traditional method of scavenging pigs to a more productive system. This requires participation of players or firms that will provide modern housing facilities, improved breeds, improved feeding systems and capacity building for farmers on recent techniques in pig production e.g., artificial insemination. The adoption of techniques that are widely used, both in Europe and America is still low in Africa, due to lack of the know-how and inadequate infrastructure. Opportunities also abound in the feed industry for supply of input or commercial feed production. Farmers, who mostly, lack the knowledge of feed formulation, rarely have access to commercial pig feed.

In most part of Africa, pigs are usually marketed live by farmers to local slaughter slab, where little or no value addition is made on the product. There is need for good investment in processing and value addition of pigs in Africa, to harness the full potential of the enterprise. Some African countries, such as Liberia and Kenya have experienced considerable growth in private institution and development partner investments in pig production and processing. Investment in research and extension services is essential to disseminate best practices, disease management strategies, and modern pig farming techniques to farmers [8,13].

### Abundance of agro-industrial by-product in Africa

As Africa’s population grows and urbanizes, there is an increasing demand for livestock products, including pork. This has heightened demand, putting pressure on the availability and affordability of conventional pig feeds. African pig farmers often rely on both global and local markets for feed ingredients. For example, the Ukraine-Russia crisis has disrupted global grain markets, affecting the availability and affordability of imported feed ingredients [32]. Meanwhile, farmer-herdsmen clashes in Nigeria and other West African countries, for instance, has disrupted local crop production, reducing the supply of locally sourced feed ingredients, resulting in severe economic repercussions. The increase in feed costs due to global grain market disruptions has strained the finances of pig farmers. Simultaneously, local conflicts have led to reduced agricultural productivity, affecting the availability of local feed ingredients and the livelihoods of smallholder farmers. The Farmer-herdsmen clashes often lead to the displacement of farming communities, where farmers are forced to abandon their farms or reduce agricultural activities due to insecurity [33]. The Ukraine-Russia crisis and farmer-herdsmen clashes in Nigeria has contributed to feed ingredient scarcity for pigs in Africa, through disruptions in both global and local markets, increased feed costs, and reduced agricultural productivity. These crises highlight the interconnections of global and regional factors in shaping the challenges faced by pig farmers in Africa in securing an adequate and affordable supply of feed ingredients. This presents an opportunity to intensify the use of agro-industrial by-products as alternative feed sources for pigs to alleviate these feed scarcity challenges. Africa’s agricultural sector generates a plethora of by-products from crops like maize, soybean, groundnut, wheat, sorghum, cassava, rice, oil palm, and sugarcane. These by-products include cassava peels, rice bran, rice husk, soybean meal, corn bran, groundnut cake, palm kernel cake, brewer’s grain, wheat offal, wheat bran, bagasse, which can serve as valuable pig feed ingredients. Many of these agro-industrial by-products are rich in energy, protein, and fiber, making them suitable for inclusion in pig diets [34]. However, proper processing and supplementation can enhance their nutritional value. Utilizing these by-products, not only reduces waste but, also contributes to a more sustainable and environmentally friendly approach to agriculture.

## CONCLUSION

Pig production is an activity that is practiced widely across Africa and has been contributing immensely to the agricultural economy of the continent, especially, as a means of livelihood for many of her rural population. In spite of the challenges facing pig production in Africa, it still has the potential to evolve into a huge contributor towards Africa’s GDP and attainment of the United Nations Sustainable Development Goals on Zero Hunger and Poverty Eradication, by providing good source of animal protein to bridge the deficit in the protein requirement for the rapidly growing Africa human population.

To harness the hidden treasure in pig production in Africa, smallholder farmers need to be provided with capacity building in the area of proper feeding and feed management, health management, biosecurity and value addition to enhance productivity and the development of the pig value-chain across Africa. Putting in place appropriate policies to favour the establishment of private pig meat processing companies or plants is critical to the development of pig production in Africa.

Sustained research efforts in the development of cost-effective feeding systems, focusing on the use of alternative feed resources or agro-industrial by-products, and development of highly adaptable breeds is critical. Careful planning and execution of disease surveillance, prevention and control measures is important for the growth of the pig industry in Africa.

## Figures and Tables

**Table 1:** Pig population and pig meat (pork) production (tonnes) in some countries and different regions of Africa

Region	Country	Population	Pig meat (tonnes)
Northern Africa			
	Algeria	4,616.00	104.50
	Egypt	11,000.00	752.04
	Morocco	7,959.00	628.65
	Tunisia	5,340.00	126.06
	Northern Africa total	28,914.00	1,610.99
Southern Africa			
	Botswana	1,345.00	225.44
	Lesotho	29,178.00	526.09
	Namibia	100,049.00	7,089.22
	South Africa	1,344,307.00	320,450.00
	Eswatini (Swaziland)	36,426.00	1,419.70
	Southern Africa total	1511304	329,710.44
Eastern Africa			
	Madagascar	1249339	19225.82
	Malawi	7,009,133.00	279,891.00
	Mozambique	1,681,933.00	121,209.04
	Rwanda	1,384,852.00	12,379.00
	Uganda	2,600,009.00	127,602.37
	Zambia	1,286,469.00	35,723.65
	Eastern Africa total	17649552	691341.7
Western Africa			
	Burkina Faso	2,693,443.00	337,410.20
	Côte d’Ivoire	430,683.00	11,588.78
	Ghana	881,909.00	29,448.31
	Nigeria	8,092,066.00	309,581.75
	Togo	1,139,962.00	13,680.20
	Western Africa total	15678263	768,766.8
Middle Africa			
	Angola	3,656,924.00	136,387.90
	Cameroon	1,926,811.00	29,349.40
	Central African Republic	1,060,125.00	19,079.04
	Democratic Republic of Congo	1,003,343.00	30,073.00
	Gabon	224,062.00	3,579.64
	Middle Africa total	8,139,402.00	223,254.73

FAOSTAT [1]; Ritchie et al [6].
